# Fluconazole - a case report on fixed drug eruption

**DOI:** 10.1186/2045-7022-4-S3-P72

**Published:** 2014-07-18

**Authors:** Magna Correia, Magna Correia, João Freitas, Rita Vieira, Filipe Perneta, Luz Brazão

**Affiliations:** 1 Internal Medicine Department – Central Hospital of Funchal – Madeira, Portugal, Portugal; 2 SESARAM, Internal Medicine, Portugal

## Background

Adverse reactions to drugs, Fixed Erythema (FE), correspond to 16-21% of all skin eruptions. This disease results in the development of one or more erythematous annular or oval, plaques as a result of systemic exposure to a drug. On reexposure relapse occurs in the same place, although new lesions may arise simultaneously in other areas. Any drug can cause FE. The physiopathology involves an allergic reaction (vasculitis).

## Methods and material

After theoretical revision and the consultation of the clinical file, the authors present a clinical case of fixed drug eruption to Fluconazole, pointing out the anamnesis, the examination and further complementary exams of diagnosis.

## Case study

Female patient of 43 years, with a past history of recurrent vaginal candidiasis, treated every time with oral Fluconazole that attended our emergency department with well demarcated erythematous plaque located on the right side of the upper lip. The patient referred that the beginning of the injuries occurred about 12 hours after taking 150 mg of fluconazole and mentioned no pain our itchy sensation. Our initial assessment was simultaneous vaginal candidiasis and herpes simplex. As so Valaciclovir was prescribed and the patient was discharged. However after the 4th day of treatment a new lesion similar to the first appeared on the dorsal part of her hand. On this new signal, the patient was diagnosed with fixed pigmented erythema to fluconazole. She stopped Valaciclovir and Fluconazole and started a topical medium power corticosteroid and a systemic antihistamine.

## Conclusion

FE has a spontaneous recovery and no complications with drug withdrawal. The use of antihistamines and topical corticosteroids relieve symptoms associated with the disease.

